# Detecting FR‐⍺ Expression Level in Cytology Effusion Specimens From Ovarian Cancer and Comparing It With Tissue Specimens

**DOI:** 10.1002/dc.70067

**Published:** 2025-12-13

**Authors:** Yadan Ma, Xiaofei Yu, Xiaohong Duan, Siqian Huang, Sishi Bai, Baizhou Li

**Affiliations:** ^1^ Department of Pathology, The Fourth Affiliated Hospital of School of Medicine, and International School of Medicine, International Institutes of Medicine Zhejiang University Yiwu China; ^2^ Department of Pathology The First People's Hospital of Yongkang Yongkang China

**Keywords:** cell block, chemotherapy, FR‐⍺, ovarian cancer, serous cavity effusion

## Abstract

**Background:**

Folate receptor‐⍺ (FR‐⍺) is an attractive target for targeted therapy. Mirvetuximab soravtansine (MIRV) has been approved by the United States Food and Drug Administration (FDA) for treating adult patients with FR‐⍺ positive, platinum‐resistant epithelial ovarian cancer, fallopian tube cancer, or primary peritoneal cancer. Therefore, identifying and detecting FR‐⍺ has become an essential part of precision medicine. This study aimed to evaluate the feasibility of detecting FR‐⍺ protein expression in serous cavity effusions using cell blocks (CBs) and compare the results with surgical pathology biopsy or resection specimens. Furthermore, we investigated potential variations in FR‐⍺ expression pre‐ and post‐chemotherapy.

**Methods:**

Immunohistochemical (IHC) staining of FR‐⍺ was performed on the serous cavity effusion specimens from 35 patients with epithelial ovarian cancer, along with matched surgical pathological (SP) biopsy or resection specimens, following the manufacturer's guidelines for IHC analysis.

**Results:**

Positive staining was observed in 30 (85.7%) and 32 (91.4%) serous cavity effusion CB and tissue samples, respectively. A total of 31 (88.5%) tissue and 28 (80%) effusion CB samples exhibited moderate‐to‐strong FR‐⍺ expression in at least 25% of tumor cells. A cutoff of 25% for FR‐⍺ expression positivity was used to demonstrate high concordance (96.4%) between cytology CBs and tissue specimens. We recommend a cutoff of 75% viable tumor cells with moderate‐to‐strong membrane staining as “positive” in CBs for greater reliability, which aligns with FDA‐approved criteria. A reasonable consistency was observed in FR‐⍺ expression between the pre‐treatment serous cavity effusion CB specimens and the new biopsy specimens obtained after multiline treatment.

**Conclusion:**

We demonstrated that serous effusion CB specimens may be effective for FR‐⍺ biomarker detection; FR‐⍺ maintains stable expression pre‐ and post‐chemotherapy, and patients can benefit from MIRV.

## 1 Introduction

Epithelial ovarian cancer (EOC) is the seventh most common cancer among women, characterized by a high mortality rate, with a 5‐year survival rate of 50% [1]. Although most patients with advanced OC are sensitive to platinum‐based chemotherapy, 80% of patients will relapse after platinum‐based chemotherapy. Almost all recurrent diseases eventually develop platinum resistance [2]. New chemotherapeutic drugs, target sites, and novel clinical advancements are urgently required to enhance efficacy in recurrent ovarian cancer. Folate receptor‐⍺ (FR‐⍺) is a potential target site for OC treatment. FR are membrane‐bound glycosyl‐phosphatidylinositol‐anchored glycoproteins encoded by the FR1 gene. They exhibit a high affinity and endocytic transport capability for their natural ligand folic acid (Vitamin B9) [3]. They are divided into FR‐⍺, FR‐β, and FR‐r. Among these, FR‐⍺ has been studied most comprehensively. The protein molecule may specifically bind to 5‐methyltetrahydrofolic acid and free folic acid, facilitating the active transport of folic acid into cells [4]. Many cancer cells overexpress FR‐⍺. Cancer cells absorb more folic acid from plasma to meet the needs of tumor growth. Additionally, they can influence cell proliferation by modulating numerous factors in the extracellular signal‐regulated kinase pathway [5]. FR‐⍺ is expressed and distributed minimally in nonmalignant tissues. However, it is overexpressed in various epithelial tumors, especially ovarian, endometrial, non‐small cell lung, triple‐negative breast, and colon cancers [6, 7, 8].

Based on this highly tumor‐restricted expression pattern, FR‐⍺ represents an attractive therapeutic target. New therapeutic drugs targeting FR‐⍺ can specifically kill cancer cells, enhance treatment efficacy, and significantly reduce off‐target effects. Mirvetuximab soravtansine (MIRV), developed by ImmunoGen, is the first targeted FR‐⍺ antibody‐drug conjugate recently approved by the US Food and Drug Administration (FDA) for treating platinum‐resistant ovarian cancer and has been included in the 2023 National Comprehensive Cancer Network Clinical Practice Guidelines [9]. This medication is indicated for adult patients with FR‐⍺ positive, platinum‐resistant EOC, fallopian tube cancer, or primary peritoneal cancer who have previously received 1–3 systemic treatments [10, 11]. During the 2023 American Society of Clinical Oncology Annual Meeting, ImmunoGen disclosed the results of a Phase 3 Clinical Trial of MIRV. This study enrolled 453 patients with FR‐⍺ positive, platinum‐resistant OC. Of the 453 patients, 227 received MIRV treatment, and 226 received chemotherapy. The efficacy results revealed that the overall response rates for the two groups were 42.3% and 15.9% (*p* < 0.001), respectively, the median progression‐free survival was 5.62 m and 3.98 m (hazard ratio [HR]: 0.65, *p* < 0.0001), respectively, and the median overall survival was 16.46 m versus 12.75 m (HR: 0.67; *p =* 0.0046). Compared with chemotherapy, MIRV is safer [2].

With certain clinical benefits indicated in OC and as a promising therapeutic target, the expression level of FR‐⍺ in OC surgical pathology has attracted continuous attention from clinicians. Therefore, FR‐⍺ identification has become essential to precision medicine. Companion diagnostic immunohistochemical (IHC) is the most commonly used detection method for FR‐⍺. IHC, as a qualitative assay, offers the benefit of direct visualization of immunoreactivity in routinely processed formalin‐fixed, paraffin‐embedded (FFPE) cancer SP biopsy or resection specimens, using commercially available antibodies to evaluate FR‐⍺ expression.

Patients with advanced OC, exceptionally high‐grade serous OC (HGSOC), the most common and fatal histological subtype of OC, usually disseminates into the peritoneal cavity, resulting in metastasis [8].

Cytological specimens may be the initial indication of a disease and the sole specimens suitable for assessing molecular diagnostic materials. However, most predictive IHC analyses are predominantly verified on surgical specimens, and only a few studies have utilized FRA‐⍺ IHC in serous cavity effusions using cell blocks (CBs) material [12, 13, 14]. The analysis of cytological specimens requires strict additional verification.

Therefore, this study aimed to evaluate the feasibility of FR‐⍺ expression in CBs derived from serous cavity effusion specimens and to compare the variations in FR‐⍺ expression levels between pre‐ and post‐chemotherapy specimens and their clinical significance. To our knowledge, no extensive studies have compared FR‐⍺ expression in cytological and SP specimens or assessed the clinical implications of FR‐⍺ expression in CBs.

## 2 Materials and Methods

### 2.1 Case Selection

With the approval of the Institutional Review Board, we obtained CB and SP specimens from patients with serous OC from the Fourth Affiliated Hospital of Zhejiang University School of Medicine and the First People's Hospital of Yongkang between January 2014 and December 2024.

This study included 35 CB specimens prepared from serous cavity effusions and their corresponding biopsy or resection specimens. The tumor cells in CBs were confirmed to originate from ovarian cancer. IHC (calretinin, WT‐1, PAX‐8, and CA125) was used to rule out metastasis from other organs. Similarly, the adequacy of CB sections stained with hematoxylin and eosin (H/E) was evaluated based on the presence of a sufficient number of tumor cells (at least 100) and normal cells, including lymphocytes, macrophages, mesothelial cells, and glandular cells. H/E‐stained CB sections analyzed were pre‐existing or obtained from CBs during the study. Cases with inadequate tumor cells in the initial H/E sections or any subsequent FR‐⍺ immunoperoxidase (IPOX)‐stained sections were excluded from the analysis. Relevant clinical data from all patients were collected, including specimen type, age, primary tumor location, stage, number of chemotherapy sessions, and cytological features.

### 2.2 CB Preparation

Cytological effusion specimens were obtained from our institution using standard techniques. The samples were freshly received in the cytology laboratory and processed within 48 h. CB was prepared using methods based on our laboratory protocol. For effusion CB preparations, 250 mL of the effusion fluid was initially divided into five 50 mL tubes and centrifuged at 377*g* for 10 min. Following the decanting of the supernatant, the sediment cell pellets from each tube were combined, and four cytospin slides were prepared (one stained with Diff–Quik and three with Papanicolaou) using one to two drops of the combined pellet. CBs were created from the combined pellet by incorporating 5 mL of 95% ethanol and 10% neutral‐buffered formalin (NBF), centrifuging the mixture at 377*g* for 10 min, and decanting the supernatant. The resultant cell pellet was removed using a spatula, wrapped in filter paper, and placed into a labeled tissue cassette. It was then immersed in 10% NBF for fixation and routine histologic processing. After paraffin embedding, 4‐μm‐thick sections were cut and stained with H/E or left unstained for ancillary tests.

### 2.3 IHC

We performed an IHC analysis of FR‐⍺, which was obtained from Suzhou Baidao Medical Technology Co. Ltd. (antibody clone number 436I6G8). We retrieved FFPE SP and CB specimens from the archives of the patients mentioned above and prepared 4‐μm‐thick sections from each sample for subsequent FR‐⍺ IHC staining.

**FIGURE 1:**
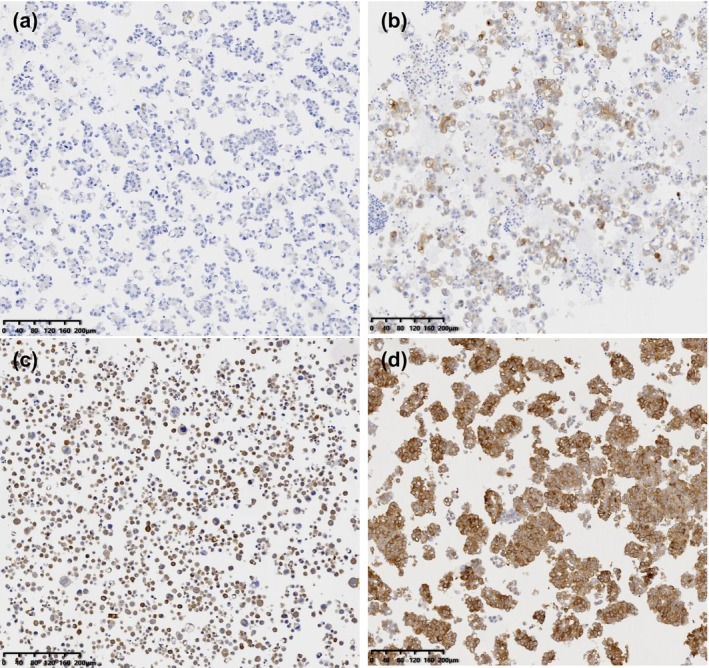
Interpretation of FR‐⍺ expression in CBs using IHC. (a–d) Intensity for negative, weak, moderate, and strong membrane reactivity staining, respectively. [Color figure can be viewed at wileyonlinelibrary.com]

A minimum of 100 viable tumor cells was required for scoring. Two pathologists evaluated the visible membranous stains and quantified the percentage of viable tumor cells in the CBs and SP specimens. All viable tumor cells were scored on a scale from 0 to 3 based on the intensity of the membrane staining, where 0 indicates no staining, 1 indicates weak staining, 2 indicates moderate staining, and 3 indicates strong staining (Figure 1). The staining area was quantified as a percentage ranging from 0% to 100%. The concordance between FR‐⍺ positivity in CBs and SP specimens was evaluated by estimating the percentage and staining intensity of matched patient samples.

FR‐⍺ expression was defined as moderate to strong membrane staining in at least 10% of viable tumor cells, as previously reported [15]. Positive and negative control experiments were performed concurrently with the FR‐⍺ experiment. According to the instructions of the manufacturer, these controls were used to validate FR‐⍺ expression, ensuring its reliability and accuracy.

### 2.4 Statistical Analysis

All tissue sections were scanned using a digital image scanner (KF‐PRO‐005, KFBIO). Statistical Package for the Social Sciences software was used for Cohen's *κ* test on the FR‐⍺ expression consistency between pre‐ and post‐chemotherapy.

## 3 Results

**FIGURE 2:**
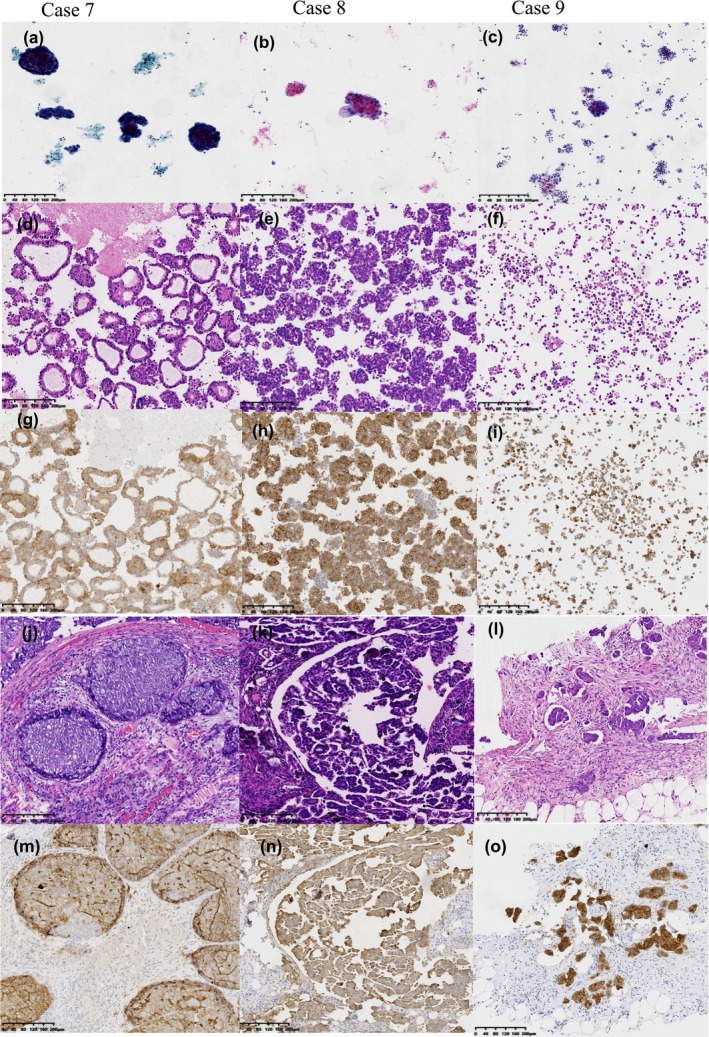
Photographs of HGSOC in serous cavity effusion. Cases 7–9 were from 3 of 35 conclusive cases. (a–c) Thin‐prep preparations present tumor cells as small, cohesive clusters with prominent nucleoli and anisonucleosis. (d–f) H/E‐stained CB sections demonstrate hyperchromasia and nuclear atypia in a glandular cavity‐like/cluster/scatter structure. (g–i) IHC staining of FR‐⍺ in cytology effusion specimens. (j–l) Relevant biopsy or resection specimens with H/E staining. (m–o) IHC staining of FR‐⍺ in tissue specimens. FR‐⍺; HGSOC, high‐grade serous ovarian cancer; H/E. [Color figure can be viewed at wileyonlinelibrary.com]

This study included 35 patients diagnosed with EOC. The mean age of the participants was 67.5 years (44–88 years). Of the 35 effusion specimens, 29 were classified as ascites, 4 as pleural effusions, and 2 as abdominal lavage fluid. In the corresponding SP specimens, 33 were obtained from primary tumor sites and 2 from metastatic lesions (including 1 case of peritoneal metastasis and 1 case of cervical biopsy). The number of viable and well‐preserved cancer cells in CBs and tissue specimens was over 100. One effusion specimen contained approximately 200 cells, and the remaining 34 effusion specimens each contained more than 500 cells. According to the International Federation of Gynecology and Obstetrics grading and staging, all 35 cases were classified as high‐grade serous carcinoma, with 12 (34.2%) at stage IIIC and 23 (65.7%) at Stage IV. In 27 effusion specimens, solitary tumor cells aggregated and spread in a clustered manner. Among them, four cases exhibited significant glandular cavity‐like formations. The remaining eight effusion specimens predominantly exhibited a pattern of single scattered cells. The cell morphology exhibited size variation, featuring large and irregular nuclei characterized by a high nuclear‐cytoplasmic ratio and prominent nucleoli (Figure 2).

All CBs and SP or biopsy samples exhibited immunoreactivity for PAX‐8, WT‐1, CK7, p53, and p16, routinely employed to determine whether the ovary is the primary tumor source. Table 1 presents patient characteristics, clinicopathological features, the number of chemotherapy treatments, and cytological features.

**TABLE 1:** Patient demographic information and clinicopathological features.

	No. (%)
Age, years	
Median (range)	67 (43–88)
Primary diagnosis (*n* = 35)	
EOC IIIC	12 (34.2)
EOC IV	23 (65.7)
Specimen source (*n* = 35)	
Ascitic fluid	29 (82.8)
Pleural fluid	4 (11.4)
Flushing fluid	2 (5.7)
Chemotherapy treatment before collection (*n* = 35)	
No treatment	
Cytological specimens	35 (100)
Tissue specimens	20 (57.1)
Treatment	
Cytological specimens	0
Tissue specimens	15 (42.8)
1 chemotherapeutic agent used	2 (13.3)
≥ 2 chemotherapeutic agents used	13 (86.7)
Cytomorphologic	
Scatter	8 (22.9)
Cluster	27 (77.1)

According to the definition of “positivity expression,” characterized by moderate‐to‐strong membrane staining in at least 10% of viable tumor cells, the positive staining identified in effusion cytology and SP specimens was observed in 30 (85.7%) and 32 (91.4%) patients, respectively. FR‐⍺ IHC results were concordant between effusion CBs and SP specimens in 33 of 35 (94.3%) cases and discordant in 2 of 35 (5.7%) cases. Of the 33 concordant patients, 3 (10%) samples yielded negative results in effusion CBs and SP assessments, whereas 30 (90.9%) samples were positive. In evaluating the staining areas of the 30 positive samples in effusion cytology, 2 (6.7%) patients had between 10% and 25% of tumor cells positive, 13 (43.3%) had between 26% and 50% of tumor cells positive, 5 (16.7%) had between 51% and 75% of tumor cells positive, and 10 (33.3%) had > 75% of tumor cells positive.

**FIGURE 3:**
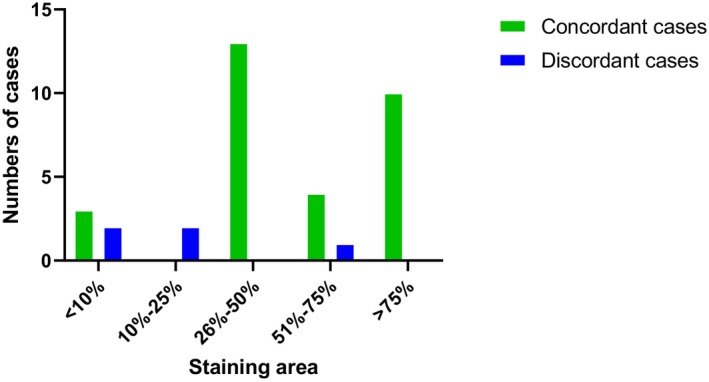
Distribution of cases based on the number of FR‐⍺ expression staining areas and concordance of staining results in CB and SP specimens. Results were concordant in 33 of 35 (94.3%) when positivity was defined as moderate‐to‐strong staining in ≥ 10% viable tumor cells. Discordance cases: Two cases had a 10%–25% staining area, and one case had a 51%–75% staining area in CBs. [Color figure can be viewed at wileyonlinelibrary.com]

Using the standard of positive staining area of FR‐⍺ tumor cells, the consistency between CBs and SP samples was 27/30 (90%). Among the three discordant cases, all were from ascitic fluid. The inconsistent staining area was distributed in the 10%–25% interval (two cases with 10%–25% of CBs against 51%–75% of SP, and one case with 51%–75% of CBs against 10%–25% of SP) (Figure 3), and no consistent cases were observed in this interval.

An upregulated cutoff of 25% for FR‐⍺‐positive staining area in CBs was utilized to demonstrate high concordance in 27 of 28 (96.4%) and discordance in one of 28 (3.57%) cases between cytology CBs and SP specimens. When an upregulated cutoff of 50% for FR‐⍺‐positive staining area in CBs was utilized, concordance was demonstrated in 14/15 (93.3%) and discordance in one of 15 cases (6.67%). The consistency markedly increased when the positive staining area cutoff was 75% (10/10 [100%]).

Considering FR‐⍺ as a therapeutic target, we analyzed FR‐⍺ expression in 15 matched specimens pre‐ and post‐chemotherapy. The average number of chemotherapy sessions was two. The basic chemotherapy regimen was the TP regimen, comprising paclitaxel and carboplatin. Cytological examination of CBs was performed before chemotherapy. After chemotherapy, histological specimens of surgical resection or metastatic foci were obtained. The time span between the collection of specimens pre‐ and post‐chemotherapy averaged 3 years.

Additionally, when the positive staining area of FR‐⍺ tumor cells was defined as a cutoff of 75%, 7 out of 15 primary CBs were positive, and 7 out of 15 histological specimens were positive after chemotherapy. The inconsistent cases in 2 of 15 (13.3%) were Cases 2 and 13 (Table 2). The overall FR‐⍺ expression consistency between pre‐ and post‐chemotherapy was 86.9%. The variation in FR‐⍺ expression pattern was statistically non‐significant pre‐ and post‐chemotherapy (*κ* = 0.733, *p* = 0.004).

**TABLE 2:** The overall FR‐⍺ expression level between pre‐ and post‐chemotherapy in 15 cases.

Patient	FR‐⍺ expression intensity and staining area (pre‐chemotherapy)	FR‐⍺ expression intensity and staining area (post‐chemotherapy)
Case 1	2+, 10%–25%	2+, 50%–75%
Case 2	3+, > 75%	3+, 50%–75%
Case 3	3+, > 75%	3+, > 75%
Case 4	3+, > 75%	3+, > 75%
Case 5	3+, > 75%	3+, > 75%
Case 6	2+, 25%–50%	2+, 50%–75%
Case 7	3+, > 75%	3+, > 75%
Case 8	1+, < 10%	1+/10%–25%
Case 9	2+, 25%–50%	2+, 50%–75%
Case 10	2+, 25%–50%	2+, 50%–75%
Case 11	3+, > 75%	3+, > 75%
Case 12	2+, 25%–50%	2+, 25%–50%
Case 13	3+, 50%–75%	3+, > 75%
Case 14	3+, > 75%	3+, > 75%
Case 15	2+, 50%–75%	3+, 50%–75%

## 4 Discussion

In this pathology era, characterized by the principle of “less is more,” cytology specimens are increasingly utilized for diagnosis and ancillary studies. Preparing a CB in cytology is a common specimen‐processing method, similar to that of an SP block, and can be useful in IPOX staining and molecular and biomarker testing [16]. Existing research has demonstrated that a high‐affinity folate receptor is present in ascitic fluid and pleural effusion [17].

Recent studies have demonstrated a high degree of consistency between cytology and surgical specimens, especially regarding markers including programmed death‐ligand 1 and CLDN18.2 [18, 19]. Few studies have utilized FR‐⍺ IHC in cytology material, with most employing surgical specimens [12, 13]. Using FR‐⍺ as a diagnostic marker for cancer in effusion cytology specimens has not been fully established. FR‐⍺ expression using the IHC technique offers the advantage of direct visualization of immunoreactivity in routinely processed FFPE tissue sections. However, the efficacy of various antibody clones that are not fully characterized and the absence of uniform criteria for interpretation and cutoff values for positive and negative cases complicate the comparison of studies.

Previous studies have used a scoring method for FR‐α‐positive expression that considers the proportion of positive cells and the staining intensity. Only samples with staining intensities of 2+ and 3+ were classified as having FR‐α positive expression [8, 20]. Currently, there is no standardized cutoff value for the tumor‐positive staining areas.

Kalli et al. used mAb 343 and established a positive cutoff value of > 1% for membrane staining in gynecological malignancies [17]. Allard et al. reported an IHC study that examined FR‐⍺ expression in gynecologic malignancies using monoclonal antibody pu17. The study employed a complex scoring system that required at least 10% of moderate or strong membrane staining [20]. A Phase I Trial demonstrated a positive correlation between FR‐⍺ expression level and the MIRV efficacy, defined by tumor cell expression levels of ≥ 25% and an IHC staining intensity of at least 2+ [21].

We initially examined FR‐⍺ expression in 35 CB from patients with advanced serous carcinomas using IHC. We defined “positive expression” as moderate to strong staining in at least 10% of viable tumor cells. FR‐⍺ expression rate was 85.7% in CBs and 91.4% in SP specimens. This finding is consistent with those of most published studies on surgically resected specimens [22].

FDA Approval Summary mentioned the VENTANA FOLR1 (FOLR1‐2.1) RxDx. The assay is a qualitative IHC test that received contemporaneous approval as a companion diagnostic device for the safe and effective use of MIRV [9]. The companion diagnostic defined the FR‐⍺ positivity as FOLR1 membrane staining of moderate and/or strong intensity in ≥ 75% of viable tumor cells. Our findings demonstrated substantial agreement between FR‐⍺ IHC performed on cytology CBs and the matched biopsy/resection specimens when a FR‐⍺ membrane staining with a cutoff of 75% viable tumor cells was evaluated.

We conclude that FR‐⍺ IHC on cytology CB specimens in HGSOC is an acceptable alternative to histological specimens for the FDA‐approved MIRV protocol. Furthermore, comparing FR‐⍺ IHC results between cytology effusion CBs and SP specimens may yield more accurate results. It may be possible to identify therapy candidates by selecting only patients with high‐intensity levels that correlate with clinicopathological data.

A major concern with the use of cytology specimens is the low cellularity of the CB. In our study, we excluded the CB with fewer than 100 tumor cells from the analysis. FR‐⍺ expression in tumors can be heterogeneous and sometimes shows striking variations between different areas of the same tumor [23]. Adequate cellularity is required to reduce the potential impact of spatial heterogeneity. Tumor cell sufficiency was defined as any number of identifiable tumor cells in the H/E and FR‐⍺ IPOX‐stained sections. The FDA approval abstract for MIRV in FR‐⍺ positive, platinum‐resistant cancers did not include a specific tumor cell number requirement [9]. According to some recommendations [23, 24, 25], to ensure the reliability of detecting IPOX expression in CB, at least 100 viable and well‐preserved cancer cells were considered adequate.

In clinical settings, targeting the folate receptor has demonstrated efficacy in preclinical studies and early clinical trials, especially for ovarian and lung cancers [4, 5, 6, 26]. The targeting mechanism of Mirvetuximab makes it a promising therapeutic option for treating these malignancies, especially in instances where conventional therapies have proven ineffective or patients present with platinum‐resistant disease [2]. Consequently, developing specific and sensitive methods for detecting FR‐⍺ expression in SP or cytological specimens is crucial. Accurate assessment of the patient's receptor status holds significant prognostic and diagnostic implications for developing FR‐⍺ selective research medications, particularly in EOC because of its molecular and cellular heterogeneity.

Patient selection for the MIRV human trial is based on IHC assessment of archival tumor tissue. This detection method evaluates the uniformity and intensity of FR‐⍺ expression and distribution pattern, with high sensitivity and specificity for distinguishing between membrane and cytoplasmic localization. However, archival tissue may inaccurately reflect FR‐⍺ expression throughout the treatment. A study involving 316 patients with OC examined the fluctuations in FR‐⍺ expression levels before, during, and after treatment. This demonstrated a decrease in FR‐⍺ levels following chemotherapy. Monitoring these changes can be a valuable tool for assessing patient responses to therapeutic interventions [27]. Other studies by Kalli et al. have demonstrated a reasonable concordance of FR‐⍺ expression in archival tissue obtained at diagnosis with data from new biopsies collected during enrollment after multiple lines of therapy [17].

Our research findings are consistent with those of the previous study by Kalli. FR‐⍺ expression is maintained in new biopsies or metastatic foci and recurrent tumors. FR‐⍺ expression levels did not vary significantly pre‐ and post‐chemotherapy. This indicates that receptor‐positive cancer cells can sustain receptor surface expression even after chemotherapy. Consequently, most patients with newly diagnosed or recurrent ovarian cancer may benefit from MIRV treatment.

IHC analysis of FR‐⍺ in CBs from serous cavity effusions can inform the decision‐making process regarding the use of FR‐⍺ targeted therapy during recurrence. As tumor heterogeneity can limit the sensitivity of IHC in detecting FR‐⍺ overexpression in CBs preparation [28, 29, 30], we recommend a cutoff of 75% of viable tumor cells with moderate‐to‐strong membrane staining as “positive” in CBs for greater reliability, which aligns with FDA‐approved criteria. However, for cases with inconsistent expression pre‐ and post‐treatment, it is necessary to expand the sample size to further investigate the reasons, and the inconsistent cases need to be studied with caution.

The FDA‐approved MIRV protocol does not specify any particular samples or fixed parameters. The VENTANA FOLR1 (FOLR1‐2.1) RxDx Assay identifies FOLR1 protein (commonly known as FR‐α and encoded by the FOLR1 gene) expression in FFPE cancer tissue specimens [9]. CBs are paraffin‐embedded cytological specimens. These versions were compared with tissues made from SP specimens of FFPE tissues. Most cytological specimens, such as fine‐needle aspiration, cavity effusion, washings, brushings, and gynecologic and nongynecologic fluid specimens, can be used to prepare CBs.

Hanley et al. [31] described preserving the antigenicity of tumor cells for accurate ICC analyses. It is crucial to use optimum fixative parameters. NBF is widely considered a universal fixative for CB preparation. NBF fixation involves immersion of cytological specimens in a neutral pH buffer containing formaldehyde. This fixation provides good preservation of cellular architecture for subsequent histological and IHC analyses [32].

Alcohol‐based fixatives, such as ethanol and methanol, are often used for cytological preparations under specific circumstances, such as Pap smears and urine samples. They are compatible with certain molecular techniques. The Nathan alcohol formalin substitute, a less toxic substitute consisting of 90% ethanol and 10% formaldehyde, is suitable for some applications. Other fixatives include formalin‐ethanol mixtures, Bouin's solution, acetic acid, and acetone, which are used in specific circumstances.

Owing to the lack of inclusion of pivotal clinical trials, adequate in‐lab validation of CB preparation, staining procedures, and pathologist scoring of CB specimens is strongly recommended to ensure agreement with histological specimens and accurate FR‐⍺ IHC scoring. To our knowledge, this study represents the largest series of cytological specimens of EOC immunostained for FR‐⍺. This provides a basis for investigating the feasibility of treatment using cytological samples. We have demonstrated the feasibility and utility of a monoclonal antibody (clone number 436I6G8) from Baidao Medical for the IHC evaluation of FR‐⍺ expression status in serous carcinomas of Müllerian origin.

The primary limitation of our research is the unavailability of corresponding post‐treatment cytological specimens. Given the high concordance in FR‐⍺ expression between cytology and tissue specimens in the previous data, we had to settle for a preliminary comparison between pre‐treatment cytological specimens and post‐treatment tissue specimens. The relatively homogeneous sample cohort and the small sample size were restricted to a single histological type of ovarian cancer. FR‐⍺ expression levels vary across different histological types of EOC [33]. Therefore, it is essential to assess whether FR‐⍺ expression is a prognostic factor in other histological types.

Because of the predominance of high‐grade advanced serous carcinoma in our EOC cohort, it was difficult to establish a correlation with tumor grade, stage, and prognosis. However, advanced tumors are more likely to express FR‐⍺ than early‐stage diseases. This limitation prevented us from investigating sensitivity, specificity, positive predictive value, and negative predictive value within the context of this study design. To our knowledge, literature assessing these parameters in the context of FR‐⍺ is nonexistent. More extensive studies are required to investigate the consistency between FR‐⍺ expression in cytological effusions and SP specimens, categorized by a single variable based on the number of tumor cells and various cutoff values.

This study represented the largest cohort of effusive fluid cytology specimens to date. However, since our study did not aim to evaluate the potential prognostic significance of FR‐⍺ overexpression, we did not draw any conclusions regarding FR‐⍺ expression as determined using IHC and its relationship with survival.

The potential value of combining targeted therapy and immunotherapy with FR‐⍺ in OC treatment requires further investigation. This approach is expected to extend the survival of patients with OC and benefit a larger population. Future studies should involve larger cohort sizes to validate and broaden the current findings, thereby enhancing the clinical relevance of FR‐⍺ expression in gynecological tumors.

## 5 Conclusion

The consistency of FR‐⍺ expression in cytological and SP samples indicates that cytological specimens can reliably identify patients with receptor‐positive tumors. This reliability makes cytological samples suitable for screening patients for targeted therapies.

## Author Contributions

Yadan Ma originally drafted, designed the study, researched the background, and critically revised the article for important information. Xiaofei Yu collated cases and critically revised the article for important intellectual content. Xiaohong Duan designed the study, researched the background, collated cases, and critically revised the article. Siqian Huang collated cases, coordinated, and critically revised the article. Sishi Bai conducted immunohistochemistry and collated cases. Baizhou Li designed the study, coordinated, reviewed, and substantively revised the manuscript for important intellectual content. All authors read and approved the final version. Each author participated sufficiently in the work and took public responsibility for appropriate portions of the content of this article.

## Funding

The authors have nothing to report.

## Ethics Statement

The research was approved by the Institutional Ethics Review Board of the Fourth Affiliated Hospital, Zhejiang University School of Medicine (YJ250401114129), and conducted in accordance with the principles of the World Medical Association's Declaration of Helsinki “Ethical Principles for Medical Research Involving Human Beings.”

## Consent

Waiver of patient informed consent was granted by the Ethics Committee.

## Conflicts of Interest

The authors declare no conflicts of interest.

## Data Availability

The datasets are fully available within the article and further inquiries can be directed to the corresponding author.
